# High-power pulsed electrochemiluminescence for optogenetic manipulation of *Drosophila* larval behaviour

**DOI:** 10.1038/s41377-025-02143-y

**Published:** 2026-02-05

**Authors:** Chang-Ki Moon, Matthias König, Ranjini Sircar, Julian F. Butscher, Ronald Alle, Klaus Meerholz, Stefan R. Pulver, Malte C. Gather

**Affiliations:** 1 Humboldt Centre for Nano- and Biophotonics, Institute for Light and Matter, Department of Chemistry and Biochemistry, University of Cologne, Cologne, Germany; 2 Centre of Biophotonics, SUPA, School of Physics and Astronomy, University of St Andrews, St Andrews, UK; 3 School of Psychology and Neuroscience, University of St Andrews, St Mary’s Quad, St Andrews, UK; 4 Institute for Light and Matter, Department of Chemistry and Biochemistry, University of Cologne, Cologne, Germany; 5 Cologne Excellence Cluster on Cellular Stress Responses in Aging-Associated Disease (CECAD), University of Cologne, Cologne, Germany

**Keywords:** Lasers, LEDs and light sources, Photonic devices

## Abstract

Electrochemiluminescence (ECL) produces light through electrochemical reactions and has shown promise for various analytic applications in biomedicine. However, the use of ECL devices (ECLDs) as light sources has been limited due to insufficient light output and low operational stability. In this study, we present a high-power pulsed operation strategy for ECLDs to address these limitations and demonstrate their effectiveness in optogenetic manipulation. By applying a biphasic voltage sequence with short opposing phases, we achieve intense and efficient ECL through an exciplex-formation reaction pathway. This approach results in an exceptionally high optical power density, exceeding 100 μW mm^−^², for several thousand pulses. Balancing the ion concentration by optimizing the voltage waveform further improves device stability. By incorporating multiple optimized pulses into a pulse train separated by short rest periods, extended light pulses of high brightness and with minimal power loss over time were obtained. These strategies were leveraged to elicit a robust optogenetic response in fruit fly (*Drosophila melanogaster*) larvae expressing the optogenetic effector CsChrimson. The semi-transparent nature of ECLDs facilitates simultaneous imaging of larval behaviour from underneath, through the device. These findings highlight the potential of ECLDs as versatile optical tools in biomedical and neurophotonics research.

## Abstract (web summary)

Very high-brightness, prolonged electrochemiluminescence is achieved by combining an exciplex-formation mechanism with biphasic pulse modulation. Together this enables optogenetic behavioural manipulation and real-time imaging of *Drosophila* larvae.

## Graphical abstract

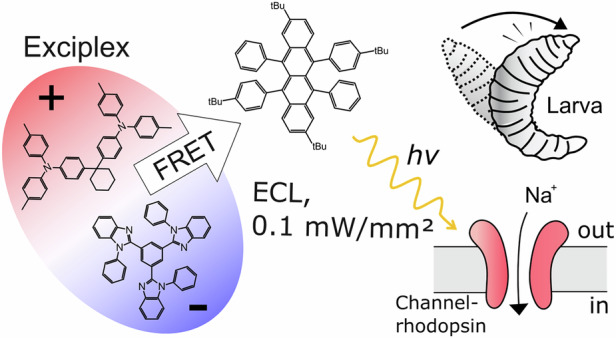

## Introduction

Electrochemiluminescence (ECL) leverages the luminescence from organic semiconductor molecules through redox reactions in (semi)fluids^1^, and this technology has proven versatile across multiple fields of biomedical research for sensing, diagnostics and in various analytical methods^2–6^. The use of fluids, including liquids^7–9^, gels^10–12^, and inks^13^, means that ECL devices (ECLDs) benefit from a much greater flexibility in device architecture than traditional solid-state devices such as LEDs, organic LEDs, and lasers. This adaptability of ECLDs makes them particularly promising as light sources for a range of applications in biomedical areas—such as optogenetics—where tailored device designs are often required for effective light delivery to both superficial and deep tissues^14–18^. Additionally, the integration of ECLDs with conductive oxide electrodes allows fabrication of highly transparent devices, thus facilitating their direct combination with microscopy.

Despite the extensive research on ECLDs, a major remaining challenge is that organic materials and solvents can be prone to irreversible side reactions, e.g., triggered by accumulation of radical ions during electrochemical processes^19^. This severely limits the feasibility of high-voltage operation, in particular operation above ±4 V, and prevents prolonged operation. Recent studies have demonstrated a luminance of 700 cd m^−2^ at ±3 V in a square-waveform AC operation using a Ru(bpy)_3_^2+^ luminophore^12^, which corresponds to an estimated optical power density (OPD) of 4.9 μW mm^−2^. While coreactant ECL enhances the light-emission process^20^, this mechanism is not suitable for light-emitting devices due to the presence of irreversible reactions. Instead, coreactant ECL has been primarily applied in biosensor devices^21^. Recently, we demonstrated exciplex-based ECL processes^22^, in which exciplex materials at high concentrations mediate redox reactions and recombination while the light emission itself is achieved by energy transfer to a fluorescent dye present at a relatively low concentration. This method offers a relatively stable reaction mechanism compared to the conventionally used ion annihilation, allowing for somewhat higher voltage operation, and thus achieving a luminance of up to 1250 cd m^−2^ and an OPD of up to 8.7 μW mm^−2^ when driven with a sinusoidal waveform with a root-mean-square voltage of 3.4 V. Beside electrochemical mechanisms, various operation strategies have been explored to mitigate the accumulation of ions, and hence to enhance the brightness and operational longevity; these include tuning AC waveforms to maintain balanced concentrations of redox species^23^, inserting a rest period between DC voltage sequences to prevent build-up of radical ions^24,25^, using current-controlled operation^25,26^, and employing a floating electrode configuration^26^.

Optogenetic experiments often require periodic light stimulation of high intensity (relative to display applications). Recent advances in channelrhodopsin development have somewhat reduced these intensity requirements, with 80 μW mm^−2^ now being sufficient for a 100% spike success rate in single-cell stimulation using ChRmine^27^. In behavioural observations involving small organisms like *Drosophila* larvae, even lower light intensities—above 10 μW mm^−2^—have elicited robust responses when using CsChrimson^28^. Therefore, reliable operation at 10 μW mm^−2^ for at least several minutes is required to facilitate effective application of ECLD for optogenetics. The most stable ECLDs reported so far, which use a floating electrode configuration and ion annihilation of Ru(bpy)_3_^2+^ luminophore^26^ have only been tested at a brightness of 114  cd m^-2^ (approximately 0.80 μW mm^−2^, i.e., 10 to 100-fold lower than required), at which they show an LT_50_ lifetime of about 80 min. Operational lifetime is known to decrease in a highly superlinear fashion, often approximately quadratically, with increasing brightness.

Here, we present the high-voltage and high-power pulsed operation of ECLDs that operate through exciplex formation and demonstrate their utility for the first time in optogenetic manipulation of the behaviour of *Drosophila* larvae. Initially, we demonstrate rapid and reversible oxidation-reduction processes for exciplex materials, performing repetitive voltage scans between ±5 V at a high rate. Following this, we apply a biphasic voltage sequence to generate rapid and intense ECL near the electrode surfaces. For ±10 V pulses of 0.2 ms duration per phase, we achieve an OPD exceeding 100 μW mm^−2^ over several thousand pulses, corresponding to 14,500 cd m^−2^. At lower-voltage operation of ±4 V, which still yields an OPD of 10.0 μW mm^−2^, corresponding to 1400 cd m^−2^, the LT_50_ lifetime of the device reaches about 43 thousand pulses, equivalent to operation duration for approximately 71 min at 10 Hz. The strong improvement in performance is achieved upon optimizing the voltage waveform, highlighting the importance of balancing ion concentrations to maximize operational longevity. Furthermore, incorporating multiple ECL pulses into a single pulse train, with short rest periods between voltage sequences, extends the duration of emission to several seconds with minimal optical power loss. Using this approach, an ECL pulse train, configured to provide quasi-continuous illumination for 4 s, elicited robust body-bending responses in *Drosophila* larvae expressing the light sensitive ion channel CsChrimson in a set of interneurons which trigger escape behaviours. By placing the larvae directly on an ECLD pixel and observing from underneath using infrared light passing through the ECLD, we can simultaneously observe the bending and twisting of larvae and deliver light pulses with high temporal and spatial resolution.

## Results

### Repeating oxidation-reduction processes

**Fig. 1:**
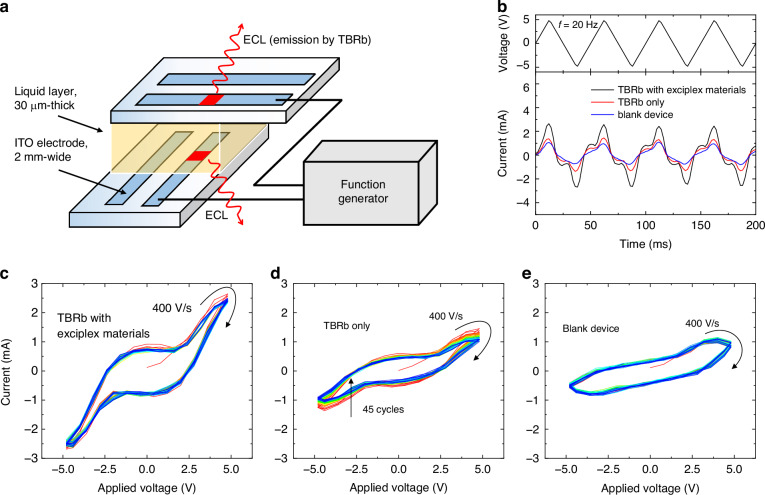
Current response to a triangular waveform voltage. **a** Schematic of the ECLD configuration. **b** Current response of three ECLDs to a triangular voltage waveform with ±5 V peak voltage and 20 Hz frequency. Voltammograms of the ECLD containing both TBRb and the exciplex materials (**c**), the ECLD containing TBRb without exciplex materials (**d**), and a blank device (**e**), each for 45 cycles with a 400 V/s ramping. Progressive cycles are indicated by red, yellow, green and blue colour. Due to the symmetry of the device, with two ITO electrodes, the resulting voltammogram curves are also symmetric

Figure 1a illustrates an ECLD in a configuration of glass(0.7 mm)/ITO(100 nm)/liquid layer(30 μm)/ITO(100 nm)/glass(0.7 mm), with an active area measuring 4 mm^2^. For our initial tests, three different solutions were used: The first solution consists of 30 mM 1,1-bis[(di-4-tolylamino)phenyl]cyclohexane (TAPC, donor for exciplex), 30 mM 2,2’,2”-(1,3,5-benzinetriyl)-tris(1-phenyl-1-H-benzimidazole) (TPBi, acceptor for exciplex), 10 mM 2,8-di-tert-butyl-5,11-bis(4-tert-butylphenyl)-6,12-diphenyltetracen (TBRb, yellow-emissive dye), and 100 mM tetrabutylammonium hexafluorophosphate (supporting electrolyte), all dissolved in a 2:1 by volume mixture of toluene and acetonitrile. The second solution contains 10 mM TBRb and 100 mM supporting electrolyte without exciplex-forming materials (also referred to as annihilation-based device). The third solution contains only 100 mM supporting electrolyte (referred to as blank device).

Figure 1b shows the current response of three different devices when subjected to a triangular voltage waveform with a peak voltage of ±5 V is applied to their ITO electrodes at a scan rate of 400 V/s (resulting in a frequency of 20 Hz). As shown in the voltammogram in Supplementary Fig. S1, the ±5 V waveform (corresponding to 10 V peak-to-peak voltage) exceeds the oxidation-reduction potential window of the TAPC-TPBi pair (2.96 V), of TBRb (2.49 V), and of the solvents (4.63 V for toluene, 6.12 V for acetonitrile^29^), indicating a voltage range that might not only drive the desired electrochemical reactions but that can also lead to electrolysis.

Comparing the average current for the first five cycles of each device reveals that the device without exciplex materials shows a 1.47-fold higher current than the blank device, while the device containing exciplex materials shows a 2.65-fold increased current compared to the blank device. This finding suggests that the ECL is significantly faster when driven via exciplex formation instead of directly by TBRb ion annihilation.

The repetitive triangular wave cycles further allow to analyse the stability of the oxidation-reduction cycles in each pathway. Figure 1c shows the voltammogram of the ECLD containing the exciplex materials, displaying swift and reversible oxidation-reduction reactions, with minimal current fluctuation over 45 voltage cycles. In contrast, the voltammogram of the annihilation-based ECLD shows clear signs of irreversibility, with a continuous reduction in peak current with increasing cycle number (Fig. 1d). Notably, the blank device did not show a significant drop in current (Fig. 1e). These observations suggest that the exciplex materials and solvent molecules are resistant to electrolysis even if a large peak-to-peak voltage is applied, at least as long as the voltage sweep is sufficiently rapid.

### Pulsed electrochemiluminescence

**Fig. 2:**
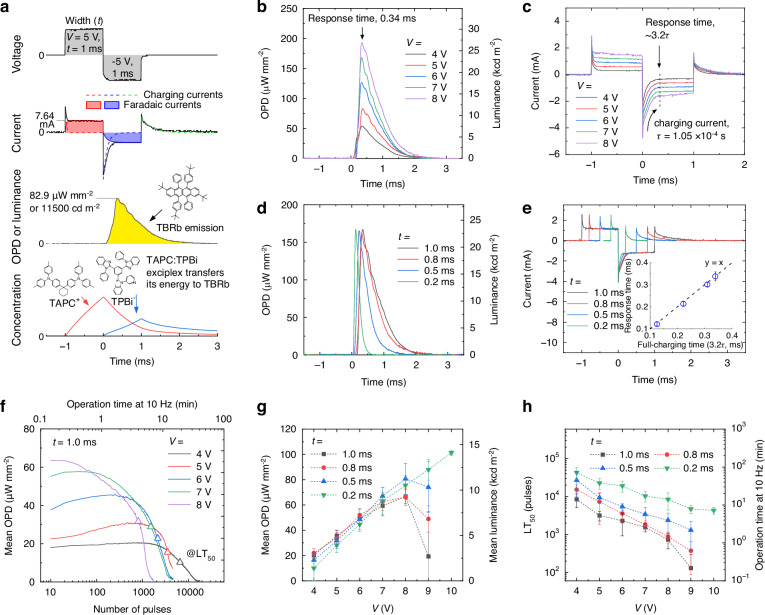
Devices operating in pulsed electrochemiluminescence (ECL) mode. **a** Shape of biphasic voltage sequence applied to an ECLD that operates based on an exciplex formation and energy transfer process, and response in current and optical power density (OPD). The bottom graph schematically shows the concentration of TAPC^+^ and TPBi^-^ ions near the electrode surface during the biphasic pulse. TAPC^+^ and TPBi^-^ ions form exciplexes in the second half of the pulse (time > 0), and subsequently transfer their energy to TBRb emitter molecules. **b** ECL and **c** current responses to biphasic pulses of different peak voltages (*V*) and with a fixed duration of 1 ms. Charging time constant (*τ*) for the second phase is 1.05 × 10^−4 ^s. **d** ECL and **e** current responses for different widths (*t*) at a fixed voltage of 7 V. Inset in (**e**) shows the linear correlation between the response time and the full-charging time (3.2*τ*). **f** Prolonged operation at a frequency of 10 Hz and at various voltages. Triangles represent the time points for LT_50_. **g** Mean OPD and mean luminance, **h** LT_50_ lifetime values of ECLDs given as the number of pulses and as the time at continuous 10 Hz operation for different peak voltages and pulse widths. Each data point is based on 4 to 6 replications, with error bars representing standard deviations

To minimize the accumulation of radical ions, thus to enable even higher-voltage operations, we applied a biphasic voltage sequence consisting of ±5 V for 1 ms in each phase at a frequency of 10 Hz to the ECLD containing exciplex materials and TBRb emitter. Figure 2a shows the resulting current density and optical power density generated by the ECLD. The current consists of faradaic currents arising from electron-transfer processes involving organic molecules and non-faradaic currents due to charging/discharging processes by mobile electrolyte ions near the electrode surfaces. These two components are represented as filled areas (faradaic) and broken lines (non-faradaic); the attribution of each component was made by simulations using an equivalent circuit model and by measuring the blank device (see ref. ^30^ and Supplementary Fig. S2). A steep rise in ECL intensity is observed during the second half of the biphasic pulse, with the emission peaking at 82.9 μW mm^−2^ and 11,500 cd m^−2^ at 0.33 ms into the second half of the pulse. The ECL intensity then gradually decreases over time for about 2 ms. The bottom graph shows a rough estimate of the concentrations of TAPC cations and TPBi anions near one of the electrode surfaces based on a simple electrochemical simulation^31^. TAPC cations accumulate during the positive voltage phase, and they react with TPBi anions generated during the negative voltage phase to form exciplexes. The exciplexes then transfer their energy to nearby TBRb molecules, resulting in the onset of light emission. At the surface of the opposing electrode, the ECL reaction occurs in response to the reverse voltage sequence, with TPBi anions generated first, followed by TAPC cations, but again resulting in exciplex formation. We attribute the small oscillation in emission during the decay in ECL intensity to various long-range coupling processes between TAPC cations and TPBi anions as these oscillations are absent in an ECLD without exciplex materials (see Supplementary Fig. S3). The ECL reaction continues even after the voltage is turned off due to the diffusion of remaining ions.

Figure 2b shows the OPD and luminance responses to biphasic pulses of different peak voltages for a fixed width of 1 ms for each phase of the pulse. As the voltage increased from 4 V to 8 V, the peak intensity increased from 54.4 μW mm^−2^ to 192.0 μW mm^−2^, corresponding to luminance values of 7.58 kcd m^−2^ and 26.9 kcd m^−2^, respectively. The shape of the ECL transient and the response time (time to peak intensity) remained consistent at  0.34 ms across different voltages. Figure 2c shows the current responses at different peak voltages. The charging current in the second phase, where the ECL process occurs, decayed rapidly with a charging time constant of 1.05 × 10^−4 ^s and remained unaffected by voltage. We found that response time aligns with 3.2*τ*; thus, the rapid charging process enables the fast response of ECLD. The faradaic process is maximized when the device is fully charged at 3.2*τ*, peaking the ECL intensity, and the process is enhanced with increasing voltage.

Next, we modified the pulse width (*t*) at a fixed pulse voltage of 7 V (Fig. 2d). As the width decreased from 1.0 ms to 0.2 ms, the response time decreased from 0.34 ms to 0.12 ms, also shortening the tail of the ECL transient. The peak intensity remained steady at approximately 167 μW mm^−2^, while the mean intensity over the duration of voltage application increased from 57.6 μW mm^−2^ to 73.2 μW mm^−2^. Figure 2e shows that a shorter pulse width shortens the charging process in the second phase, while maintaining a linear correlation between the response time and the full-charging time (3.2*τ*, inset). Given that the faradaic current after charging remained consistent, the peak ECL intensity was not affected by the pulse width.

Figure 2f shows prolonged pulsed operation at a frequency of 10 Hz. The LT_50_ parameter is defined as the number of pulses applied until the mean OPD reduces to 50% of its maximum; this value is represented by triangles in Fig. 2f. Higher voltages accelerated degradation due to the increased likelihood of undesired side reactions within the liquid layer.

Figure 2g summarizes the mean OPD and mean luminance measured at various voltages and pulse widths. A pulse width longer than 0.5 ms resulted in operation failure at *V* > 9 V, with reduced OPD values and higher variations between measurements, due to significant device degradation. In contrast, operation at *t* = 0.2 ms allows for the voltage to be increased to up to 10 V, resulting in a maximum mean OPD of 101 μW mm^−2^ and a luminance of 13.9 kcd m^−2^. Figure 2h summarizes the LT_50_ lifetime values of our ECLDs given in terms of the number pulses and the operating time at a pulsing frequency of 10 Hz (graphs for OPD vs number of pulses are shown in Supplementary Fig. S4). As expected, the lifetime improves when reducing the voltage and pulse width. Under conditions corresponding to the maximum achievable mean OPD (101 μW mm^−2^, reached at *V* = 10 V, *t* = 0.2 ms), LT_50_ was 4304 pulses and 7.2 min. For a mean OPD of 10.0 μW mm^−2^ and a luminance of 1400 cd m^−2^ (*V* = 4 V, *t* = 0.2 ms), the longest operational LT_50_ was achieved, totalling 42,880 pulses and 71.5 min.

### Modulation of the waveform

**Fig. 3:**
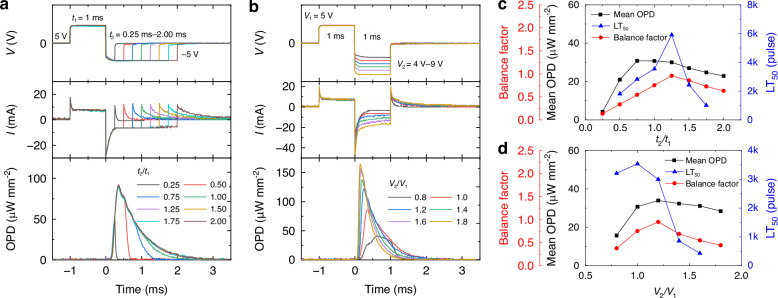
Modulation of ECL pulse. **a**, **b** ECL responses to biphasic voltage sequences with varying relative pulse widths (*t*_2_/*t*_1_) and voltages (*V*_2_/*V*_1_) for the first and second phase of the pulse. The voltage and width during the first phase are fixed to 5 V and 1 ms, respectively. **c**, **d** Balance factor for faradaic current, mean OPD, and LT_50_ as a function of the *t*_2_/*t*_1_ and *V*_2_/*V*_1_ ratios, respectively

As shown in Fig. 2a, power consumption through the charging/discharging processes is larger in the second voltage phase than in the first, indicating an unbalanced supply of ions and counterions. To mitigate this problem, we further modulated the applied waveform by testing asymmetric biphasic pulses. Figure 3a shows the current densities and ECL intensities obtained in response to ±5 V pulses with a fixed width of the first phase (*t*_1_ = 1.0 ms) and varying widths of the second phase (*t*_2_). Increasing the relative width *t*_2_/*t*_1_ extended the faradaic current during the second phase. While the width of the second phase determined the tail length of the ECL transient, it did not affect the ECL peak intensity or response time when *t*_2_ was sufficiently long for charging, i.e., when *t*_2_ > 0.34 ms. The ECL tail length increased up to *t*_2_/*t*_1_ = 1.25, suggesting an ionic balance at this pulse ratio. Figure 3b shows analogous measurements with a fixed voltage during the first phase of the pulse (*V*_1_ = 5 V) and varying voltage in the second phase (*V*_2_), with each pulse phase lasting 1 ms. Increasing the relative voltage *V*_2_/*V*_1_ resulted in higher negative faradaic currents, higher ECL peak intensities, and shorter response times by charging faster, demonstrating that the voltage during the second phase directly affects the overall ECL dynamics.

To quantify the balance between the positive and negative faradaic currents, we introduce a balance factor for the faradaic current, defined as:

$${\rm{Balance\; factor}}=\frac{\min \left({I}_{{\rm{pos}}},{I}_{{\rm{neg}}}\right)}{\max \left({I}_{{\rm{pos}}},{I}_{{\rm{neg}}}\right)}$$

where *I*_pos_ and *I*_neg_ are the time-integrated faradaic currents during the positive and negative pulses, respectively. A balance factor of 1 represents perfectly balanced current injection, i.e., an ionic balance during device operation. Figure 3c, d summarize the key device characteristics—balance factor, mean OPD, and LT_50_—as functions of *V*_2_/*V*_1_ and *t*_2_/*t*_1_ ratios, respectively. The ionic balance achieved at *t*_2_/*t*_1_ = 1.25 maximizes operational longevity, while an excessive supply of counterions at *t*_2_/*t*_1_ > 1.25 significantly reduces the LT_50_ value. In contrast, the mean OPD value was highest at *t*_2_/*t*_1_ = 0.75 because ECL reactions occurring near the electrode surfaces are more efficient than those occurring due to ion diffusion at a later time point. In contrast, balancing the ion concentrations by increasing the voltage in the second phase did not improve the operational longevity, presumably because the increased likelihood of side reactions at higher voltages outweighs the benefits of ion balancing. As a result, the optimal LT_50_ value is achieved at *V*_2_/*V*_1_ = 1.0.

**Fig. 4:**
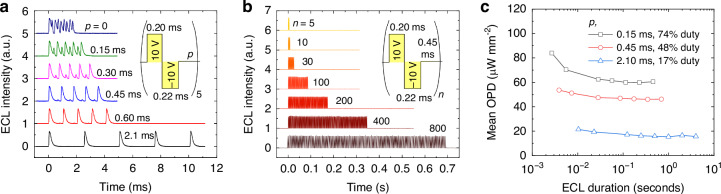
Pulse train consisting of multiples ECL pulses. **a** Five ECL pulses with varying rest periods (*p*) between biphasic voltage pulses. **b** ECL pulse trains incorporating between *n* = 5 and *n* = 800 biphasic pulses with a rest period of 0.45 ms between pulses. **c** Mean OPD as a function of number of applied voltage pulses for different rest periods

Next, we incorporated pulse trains consisting of multiple pulse sequences to achieve prolonged ECL at high optical power, as is required for the intended application in optogenetics. We optimized a rest period (*p*) between sequences to prolong ECL by refreshing the concentration distribution within the liquid layer. Figure 4a shows the ECL response to five subsequent voltage pulses, each at ±10 V and with *t*_1_ = 0.20 ms and *t*_2_ = 0.22 ms to ensure balanced ion concentrations as per our previous optimization (see Supplementary Fig. S5). The rest period varied from 0 to 2.1 ms. Without rest periods (*p* = 0), ECL peaks appeared during every pulse phase due to the presence of residual ions, and this caused the peak intensity to progressively deteriorate. As the rest period increased in duration, the ECL peaks corresponding to individual biphasic sequences became more distinct, with stable ECL intensity achieved at *t* ≥ 0.45 ms (see Supplementary Fig. S6 for the correlation between peak intensity and rest period). Figure 4b demonstrates that high intensity ECL was maintained for over 0.7 s without significant reduction in ECL intensity by incorporating *n* = 800 pulse sequences with a 0.45 ms rest period.

Figure 4c shows the mean OPD for a single pulse train with increasing number of pulses, incorporating rest periods of 0.15 ms, 0.45 ms, and 2.10 ms, corresponding the duty ratios of 74%, 48%, and 17%, respectively. A rest period of 0.45 ms achieved intense and stable light output, yielding a mean OPD of 46.2 μW mm^−2^ over 0.7 s at *n* = 800 pulses with minimal optical losses introduced with increasing *n*. A longer rest period of 2.10 ms allows for more pulses before device degradation becomes significant, resulting in a mean OPD of 15.6 μW mm^−2^ over 4.0 s at *n* = 1600 pulses. This configuration is suitable for applications requiring extended illumination periods, albeit with a lower mean OPD compared to the shorter rest periods. Conversely, a shorter rest period of 0.15 ms produces more intense ECL pulses, with an OPD of 60.4 μW mm^−2^ at *n* = 800. However, the insufficient removal of radical ions led to a gradual decrease in OPD with increasing number of pulse sequences, ultimately resulting in unstable operation.

### Optogenetic stimulation of *Drosophila Larvae*

**Fig. 5:**
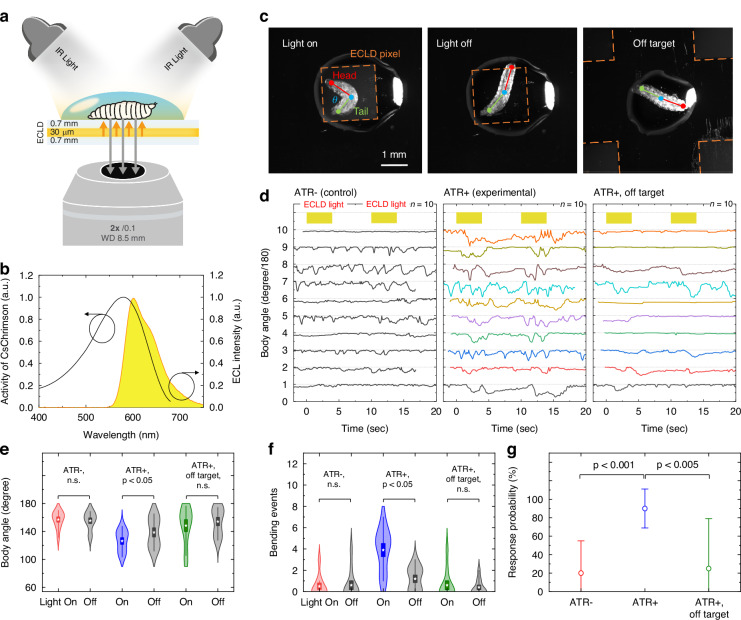
Optogenetic manipulation of *Drosophila* larvae behaviour. **a** Schematic of the inverted microscope setup. **b** ECL emission spectrum (yellow) and activity spectrum of CsChrimson (black line). **c** Illustration of the three body points (red, blue, green) tracked in larvae to quantify their behavioural response to ECLD illumination, indicated for animal placed on an ECLD pixel with light on (left) and light off (centre), as well as for animal placed away from the active pixel, i.e., off target (right). **d** Body angle over time for 10 individual larvae placed on an ECLD pixel for an ATR- control (left) and ATR+ experimental animals, i.e., fed with ATR supplemented food (centre), as well as for off target ATR+ animal (right). Light stimulation and darkness periods are 4 seconds and 6 seconds, respectively. **e**, **f** Statistical analyses of body angle and bend events for larvae in experimental and control groups as well as off target group during light “on” and “off” phases. **g** Response probability in control and experimental groups, highlighting the likelihood of bending in response to ECL light stimulation

To show the potential value of ECLDs and the new driving scheme developed here for biomedical and neurophotonics research, we examined the ability of ECLDs to optogenetically activate neurons in in *Drosophila* larvae, which in turn trigger an escape behaviour in the animals. To do this, we placed first or second instar larvae within water droplets of approximately 3 mm diameter located on the ECLDs as illustrated in Fig. 5a. The water droplets allowed us to confine the larvae to the position of an ECLD pixel. We utilized a transgenic *Drosophila* line expressing CsChrimson^32^, a light gated cation channel with an excitation spectrum closely aligned with the ECL emission spectrum (Fig. 5b). In this genotype, light pulses activate ‘Down and Back’ (DnB) interneurons^33^, which cause the larvae to bend and roll away in response to perceived threats. Behavioural responses of 10 individual larvae per group were recorded with an inverted microscope, leveraging high transmittance of the ECLD in the range of visible light-near infrared (see Supplementary Fig. S7). To observe larval behaviour, animals were illuminated with infrared light (*λ*_peak_ = 853 nm, Supplementary Fig. S8), which is outside the excitation wavelength range of CsChrimson, and which can pass through the ECLD without substantial loss. In the absence of ECLD light, the larvae generated peristaltic waves, performed head sweeps, and turned within the water droplet, all characteristics of normal exploratory behaviour (see Supplementary Videos S1-2).

To test the behavioural response upon optogenetic stimulation, we tracked the angle between head and tail of the larvae (referred to as “body angle” $\theta$ in Fig. 5c) during 25-s-long trials which consisted of alternating 4-second light “on” and 6-second light “off” phases. During the “on” phases, the ECLD operated at a mean OPD ranging from 1.84 µW mm^−2^ ( ± 4 V, *n* = 1600, *p* = 2.10 ms), which is near the light sensitivity threshold of CsChrimson in *Drosophila* larvae (2.0 µW mm^−2^ at 90% response probability, as estimated by Murawski et al.^28^), to 15.6 µW mm^−2^ ( ± 10 V).

Functional expression of CsChrimson requires the presence of all-trans-retinal (ATR) as co-factor. When food was not supplemented with ATR (ATR- control group), ECLD light pulses did not induce significant changes in body angle (Fig. 5d). In contrast, larvae in the ATR+ experimental group displayed a marked decrease in body angle during the “on” phases compared to the “off” phases. The average body angles during “on” and “off” phases were measured at 126° and 139°, respectively (Fig. 3e, *p* < 0.05). Note that the onset of the larvae response was often delayed, and the reduced body angle persisted after the ECLD was turned off, which was included in the “off” phase statistics, indicating that effects may be even stronger than reported here.

Trials conducted with larvae positioned approximately 2.1 mm to the side of the active pixel (“off-target” trials, Fig. 5c) did not show significant changes in the body angle, with average body angles of 148° and 153° during the “on” and “off” phases, respectively (see Supplementary Videos S3–5). These results confirm that ECLDs can elicit an optogenetic response with high spatial resolution, while also corroborating that behavioural responses are due to light stimulation rather than by any electrical, chemical, or thermal effect (see Supplementary Fig. S9) from device operation.

Further analysis involved counting the number of “bend” events, where the body angle dropped below 90 degrees for at least 100 ms during the “on” phase. Larvae in the experimental group showed a highly increased number of bend events during the “on” phases compared to the “off” phases (Fig. 5f, *p* < 0.005). In contrast, neither the off-target group nor ATR- control group showed any significant differences between light “on” and “off” phases.

To quantify the likelihood of larvae reacting with a bend event to ECLD light, we calculated the response probability of each larva as the ratio of stimulations evoking a behavioural response, to the total number of stimulations (see Methods for response criteria). The ATR- control group set a baseline probability of 19% due to spontaneous movement. In contrast, larvae in the experimental group had a 90% probability of reacting to the ECL pulses (Fig. 5g, *p* < 0.001). The response probability of the off-target group was not significantly different from the baseline of the control group.

## Discussion

Due to insufficient light output and low operational stability, ECLDs have so far faced challenges in lighting applications. This study demonstrated a significant improvement in ECL intensity and lifetime through a thorough optimization of the pulsed operation of ECLDs. A robust electrochemical operating mechanism based on exciplex formation on the molecules TAPC and TPBi facilitated the application of voltage up to 10 V in the pulsed operation. These improvements were applied to highlight the potential of ECLDs as a light source capable of delivering stable, high-power light emission for biomedical research, in particular for optogenetics experiments.

Intense ECL pulses were generated through the use of an exciplex-based reaction pathway followed by energy-transfer to a terminal emitter and applied biphasic voltage sequences to the device that consisted of pulses with opposing voltage lasting of order 1 ms each. We observed a rapid increase in ECL intensity during the second half of each biphasic sequence arising from a reaction of counter ions with ions accumulated during the initial phase of the voltage pulse. The subsequent gradual decrease in intensity over time was attributed to slow ion diffusion. The fast ECL pulse response, occurring within 0.34 ms, was driven by the rapid charging process. Pulse widths shorter than 1 ms further reduced the response time, with a linear correlation of 3.2 times the charging time constant. We measured an exceptionally high mean OPD of 63.4 μW mm^−2^ with a peak OPD of 192 μW mm^−2^ in an ECL pulse for a biphasic voltage sequence of ±8 V and 1 ms duration. Reducing the pulse width allowed for a further increase in voltage, achieving an even higher mean OPD of 101 μW mm^−2^ at ±10 V and 0.2 ms pulse duration; 4304 emission pulses were delivered in this configuration until 50% degradation.

A modest extension of the width of the second phase of the biphasic voltage sequence was found to effectively increase the operational lifetime through balancing ion concentrations. Increasing the voltage during the second phase of the biphasic pulse enhanced the mean OPD and shortened the response time but was not effective in increasing the longevity due to enhanced side reactions.

Finally, we incorporated pulse trains consisting of multiple optimized voltage sequences to extend the duration of ECL of high optical power. Inserting a rest period longer than 0.45 ms refreshed the device between sequences, allowing for more than a thousand ECL pulses without noticeable device degradation. As a result, a single pulse train maintained stable ECL pulses at an optical power of 60.4 μW mm^−2^ for 0.46 s using a 0.45 ms rest period, and at an optical power of 15.6 μW mm^−2^ for 4.0 s using a 2.10 ms rest period.

To demonstrate their potential for biomedical applications, we used ECLDs to optogenetically stimulate and observe *Drosophila* larvae. The light output of the ECLD operating by optimized pulse sequences effectively and reliably induced escape behaviours in larvae expressing CsChrimson in an ensemble of neurons. When exposed to alternating 4-second light “on” phases and 6-second light “off” phases, the larvae exhibited a significantly reduced body angle due to a body-bending behaviour during the “on” phases (126°) compared to the “off” phases (139°). This was in line with the first stage of rolling behaviour observed by Burgos et al.^33^. Larvae in the experimental group demonstrated a striking 90% response rate to optogenetic stimulation, significantly higher than the 19% baseline observed in the control group. In addition, the larvae in the experimental group showed negligible response to light when they were positioned away from the ECLD pixels, demonstrating the high spatial resolution of optogenetic triggering using ECLDs.

Optogenetics is a widely used tool for studying the nervous system across multiple model organisms and fields of biomedical sciences. However, traditional light sources make imaging during optogenetics experiments challenging as the opaque nature of most light sources requires careful placement of the microscope or camera away from the light source or use of multiple light paths and/or dichroic mirrors and filters. Our semi-transparent ECLDs open up new opportunities by providing the ability to image through the light source, placing the microscope or camera directly in line with the animal or tissue of interest. In addition, the light source can be placed directly in contact with the target animal or tissue if needed, thus opening a broad range of experimental configurations for optogenetics, biomedical research and neurophotonics in general. Overall, these experiments show the potential for ECLDs to not only be an effective illumination tool in optogenetics but to provide a platform for a range of experiments not possible with conventional LEDs.

In order to achieve spectral alignment with the diverse activation spectra of different channelrhodopsins, it will ultimately be important to realize ECLDs emitting light of different colours. Exciplex-based ECLDs offer the potential to accommodate terminal emitters with a range of different emission spectra as excitation of the terminal emitter occurs via FRET, thereby avoiding the constraints in selecting emitter materials that are associated with redox and ion-annihilation processes of the emitter. However, to realize this potential, spectral engineering of exciplexes will be essential in order to ensure sufficient overlap between exciplex emission and emitter absorption and thus guarantee an effective FRET process.

## Materials and methods

### Materials

TAPC, TCTA, and TBRb were purchased from Luminescence Technology Corp. and used as received. Anhydrous toluene, anhydrous acetonitrile, and TBAPF_6_ were purchased from Merck KGaA. ITO-coated glass substrates were purchased from Xinyan Technology Ltd. The encapsulation glue (3035BT) was purchased from Threebond International.

### Device fabrication

A schematic of the fabrication procedure for an ECLD is given in Supplementary Fig. S10. To manufacture an ECLD, a pair of rectangular glass substrates with a size of 24 mm by 15 mm, each coated by two 2 mm-wide ITO layers, were cleaned by immersion in a detergent solution (2% Hellmanex® III in Milli-Q water) and subsequently in isopropyl alcohol. The cleaned substrates were dried on a hot plate. The surfaces of ITO were treated with UV-ozone for 15 min. The glass substrates were bonded together at an angle of 90°, where the ITO surfaces of the two substrates faced each other and intersected with an overlapping area of 4 mm^2^. A UV-curable NOA68 resin (Norland Products) was applied as droplets to corners of the glass substrate avoiding contact with the active areas and remaining the edges of the bonded substrate open for liquid injection. Polystyrene microbeads were mixed in the resin to keep the gap between glass substrates at 30 μm. The bonded substrates were then transferred to a nitrogen-atmosphere glove box. 6.5 µL of ECL solution was pipetted into gap between substrates for capillary filling. The edges of the liquid-filled area were sealed by 3035BT (Threebond International) resin and then cured under UV light. Consequently, an ECLD with four active areas operating independently is produced.

### Device characterization

To characterize the electroluminescence properties of the devices, we employed an arbitrary waveform generator (33220 A, Agilent Technologies) that allowed to drive the devices with biphasic rectangular pulses, varying in frequency, voltage, and pulse width for both the positive and negative voltage parts as described in the main text. The waveform was programmed via the SCPI interface using a custom Python software. During the pulsing, the device current was measured by recording the voltage drop across a 1 Ohm shunt resistor in series with the ECLD, using an oscilloscope (Rigol DS1000Z). The ECL transient was monitored using a calibrated silicon photodiode (PDA100A2, Thorlabs), which was connected to the second channel of the same oscilloscope for temporal readout. The ECLD was positioned in the centre of a 55 × 55 cm dark box in a temperature-controlled room. The photodiode was positioned 168 mm from the device on one side, while the emission from the opposite side of the device was disregarded in the analysis. Spectral data were obtained using a fibre-coupled spectrometer (Ocean HDX, Ocean Insight) and used to convert the photodiode reading into absolute units in watt and candela, taking into account the distance between the device and the photodiode and the geometry of photodiode, and assuming a Lambertian emission profile for the ECLD. The optical power density and luminance calculation considered the emissive area of 4 mm^2^. The general procedure for measuring optical power density and luminance with a calibrated photodiode and a fibre-coupled spectrometer follows the process routinely used in our laboratory for characterization of OLEDs^34^. For lifetime measurements, the ECL transient was recorded at one-second intervals over extended durations.

Cyclic voltammograms in Fig. 1b–e were obtained by recording the voltage drop across a 1 Ohm shunt resistor, connected in series with the device, while applying a triangular voltage created by the waveform generator to each device.

### Drosophila culture

Transgenic *Drosophila* flies with 412-GAL4 (a.k.a DnB-GAL4) stably combined with 20XUAS-IVS-CsChrimson coupled to a mVenus fluorophore were used for all experiments. 412-GAL4 expresses in DnB interneurons, which trigger larval escape behaviours when activated. Animals were raised in vials on standard cornmeal-based food at 20 °C on approximately 12:12 light-dark cycles. Two weeks before the optogenetic experiments, eggs were transferred onto fresh vials with 5 ml media and 0.725 mM all-trans retinal (Sigma Aldrich, USA) final concentration. Vials were kept in the dark at 20 °C and away from heat. ATR- control groups for the optical stimulation were 412-GAL4; 20XUAS-IVS-CsChrimson-mVenus larvae, kept under the same conditions as the optogenetic group, but without ATR supplemented food.

### Drosophila imaging

Imaging was performed on a standard inverted microscope (Tie2, Nikon, Japan) using a Plan Apo 2x objective with a numerical aperture of 0.1 and a working distance of 8.5 mm (Nikon, Japan). Two infrared light sources (IR49S, Andoer, China) were installed above the microscope stage to illuminate the larvae. Videos with 2048 by 2048-pixel resolution, 20 frames per second and 16 bit depth were recorded with an sCMOS camera (Orca Flash 4.0, Hamamatsu, Japan) controlled by the NIS Elements Suite (Nikon, Japan). First or second instar larvae were picked from the vials under low ambient light, briefly washed in water and transferred into a drop of water on the surface of the ECLD above an active pixel. The larvae were habituated for two to three minutes without ECLD light before the experiment was started. After the stimulation of the larva on top of a pixel was completed, the same larva was moved to an off-pixel area with a fresh drop of water and the experiment was run again after a habituation period. ATR- control larvae were only imaged on pixels instead of both on and off pixels. Larvae were only partially submerged for 5-10 min in any given experiment and had periodic access to air through posterior spiracles.

Device heating was checked with a thermal camera (C2, FLIR Teledyne) and data was analysed by averaging the reading in the thermal images over the emissive area of the ECLD, measuring 2 × 2 mm².

### Data analysis and statistics

Videos were analysed using the manual tracking plugin of ImgeJ2 (Version 2.14.0). Further analyses were performed with custom scripts in Matlab 2023a (MathWorks) and Origin 2024 (Origin Lab). The head, tail and centre of the larvae were tracked and the body bending angle was calculated using the following equation:

$$\cos \theta =\frac{\overrightarrow{{\rm{head}}}\times \overrightarrow{{\rm{tail}}}}{\overrightarrow{\left|{\rm{head}}\right|}\times \overrightarrow{\left|{\rm{tail}}\right|}}$$

All statistical testing was done using two samples t-tests with an alpha of 5%. The overall body during light on and off phases were compared within each experimental group (ATR-, ATR+ , ATR+ , off target). To analyse specific behavioural responses to light stimulation, body-bending events per light on and off phase per trial were counted. A body-bending event was automatically detected by thresholding during data analysis when the body angle *θ* was below 90° for a minimum of 100 ms and compared within each experimental group. To further correlate a body-bending event to a light on phase, a response probability was calculated based on the ratio of responses to stimulations during light on phases and compared between groups.

## Supplementary information

Supplementary Information

Supplementary Video 1

Supplementary Video 2

Supplementary Video 3

Supplementary Video 4

Supplementary Video 5

## Data Availability

The research data supporting this publication can be accessed at 10.17630/4ffc6f6f-7d04-43ed-9ed1-9d1002f0b775.
