# Supplementary material for: High-power pulsed electrochemiluminescence for optogenetic manipulation of Drosophila larval behaviour
Source: Light Sci Appl. 2026 Feb 5;15:104. doi: 10.1038/s41377-025-02143-y (PMC12873326; doi:10.1038/s41377-025-02143-y)
Supplement: Supplementary file 1 — Supplementary Information [file 41377_2025_2143_MOESM1_ESM.docx]

**Supplementary Information for**

High-power pulsed electrochemiluminescence for optogenetic manipulation of Drosophila larval behaviour

Chang-Ki Moon^1,2^, Matthias König^1,2^, Ranjini Sircar^1,3^, Julian F. Butscher^1,2^, Ronald Alle^1^, Klaus Meerholz^1^, Stefan R. Pulver^3^, Malte C. Gather^1,2,4^

^1^Humboldt Centre for Nano- and Biophotonics, Institute for Light and Matter, Department of Chemistry and Biochemistry, University of Cologne, Greinstr. 4-6, 50939 Cologne, Germany

^2^Centre of Biophotonics, SUPA, School of Physics and Astronomy, University of St Andrews, North Haugh, St Andrews KY16 9SS, United Kingdom

^3^School of Psychology and Neuroscience, University of St Andrews, St Mary's St Mary's Quad, South St, St Andrews KY16 9JP, United Kingdom

^4^Cologne Excellence Cluster on Cellular Stress Responses in Aging-Associated Disease (CECAD), University of Cologne, Cologne, Germany

**Supplementary Video S1 Behavior of a second instar Drosophila larva trapped within a water droplet without light stimulation**

**Supplementary Video S2 Behavior of a first instar Drosophila larva trapped within a water droplet without light stimulation**

**Supplementary Video S3 Trial using control larva (ATR-) on ECLD pixel.**

**Supplementary Video S4 Trial using experimental larvae (ATR+) larva on ECLD pixel.**

**Supplementary Video S5 Trial using experimental larvae (ATR+) larva off ECLD pixel.**

**
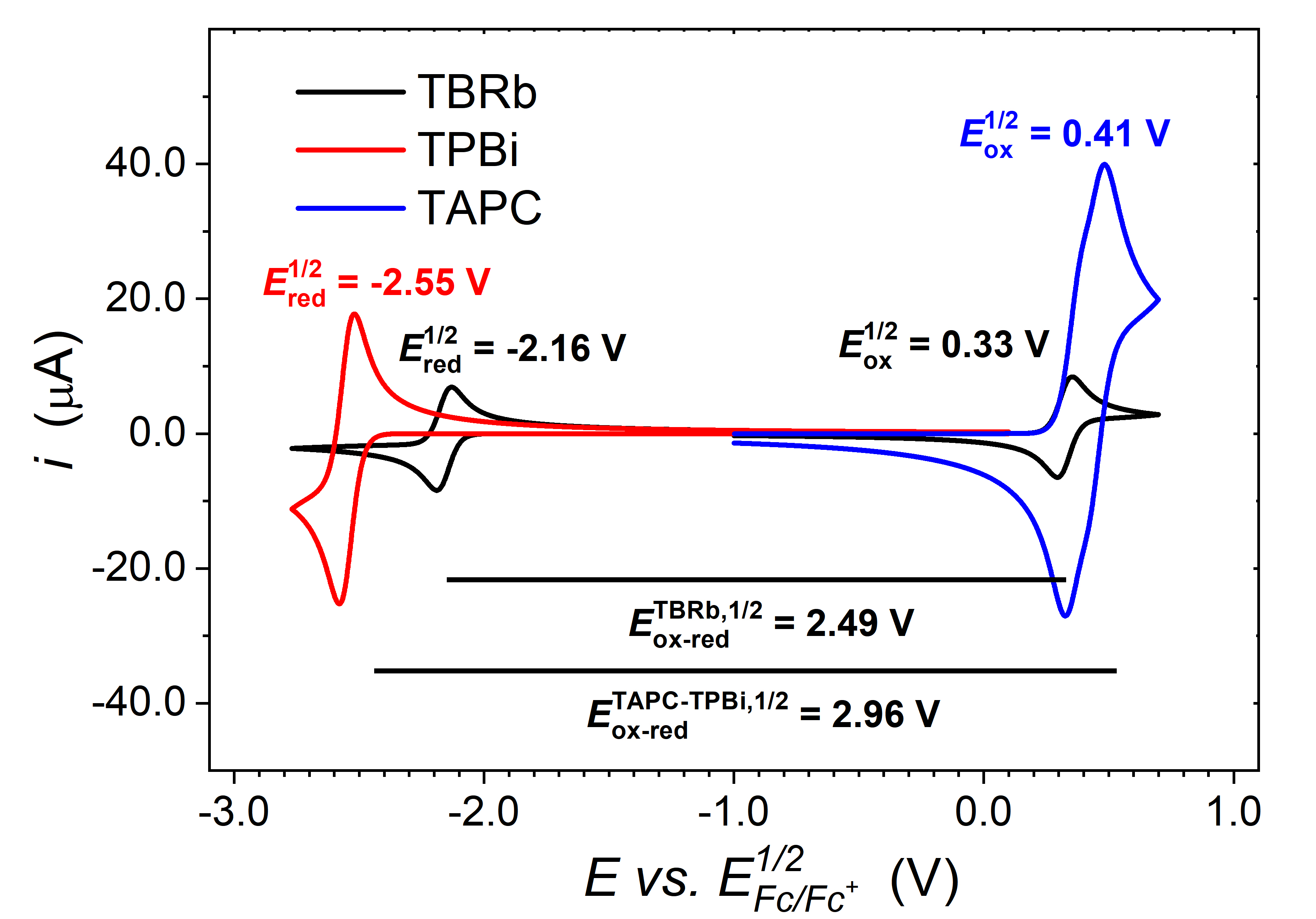
**

**Fig. S1 Cyclic voltammograms for TBRb, TPBi, and TAPC.** Cyclic voltammetry was performed using a solvent mixture of toluene and acetonitrile in a 2:1 ratio. The concentration of the supporting electrolyte, TBAPF_6_, was 0.1 mM, and the concentration of each compound was 1 mM. The measurement used a non-commercial airtight glass cell, in which a Luggin capillary separated the working and reference electrodes. The cell was maintained in an argon atmosphere. A glassy carbon electrode tip (*d*=2 mm) from Autolab/Metrohm, a platinum wire, and a pure silver wire were used as the working electrode, counter electrode, and reference electrode, respectively. The scan rate was set to 0.1 V/s. All potentials are given relative to the half-wave potential of the ferrocene/ferrocenium redox couple.

**
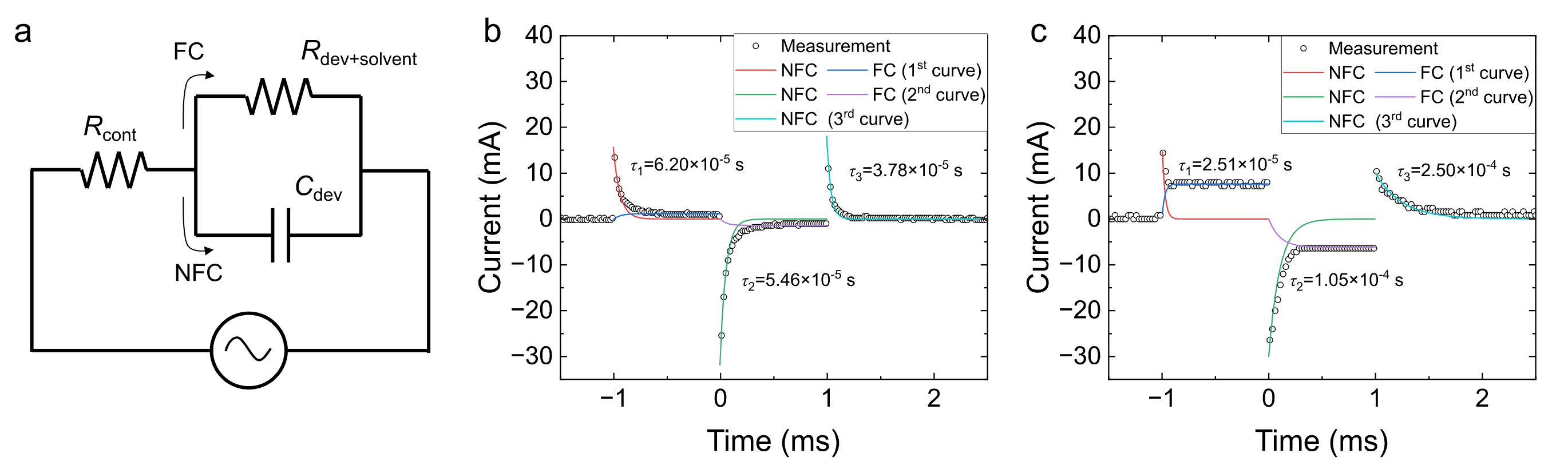
**

**Fig. S2 Modeling of the faradaic current (FC) and non-faradaic current (NFC**). **a** Equivalent circuit of ECLD. *R*_cont,_ *R*_dev+solvent_ and *C*_dev_ represent the contact resistance, device resistance originating from faradaic processes of dissolved organic materials and solvent molecules, and device capacitance, respectively. **b** Simulated FCs and NFCs for a blank device only containing an electrolyte and **c** an ECLD that additionally contains exciplex materials and TBRb emitter, both operated by a biphasic sequence with a voltage of 5 V and a width of 1 ms. The exponential decay time constants (*τ*’s) of the current after voltage application, reversal, and termination are 6.20×10^-5^ s, 5.46×10^-5^ s, and 3.78×10^-5^ s, respectively, for the blank device; and 2.51×10^-5^ s, 1.05×10^-4^ s, and 2.50×10^-4^ s, respectively, for the ECLD. The fact that comparable time constants were observed in these two devices indicates that the NFCs are governed by charging and discharging currents. The Faradaic currents in the blank device are small compared to the ECLD and arise from solvent molecules activated by voltage application.


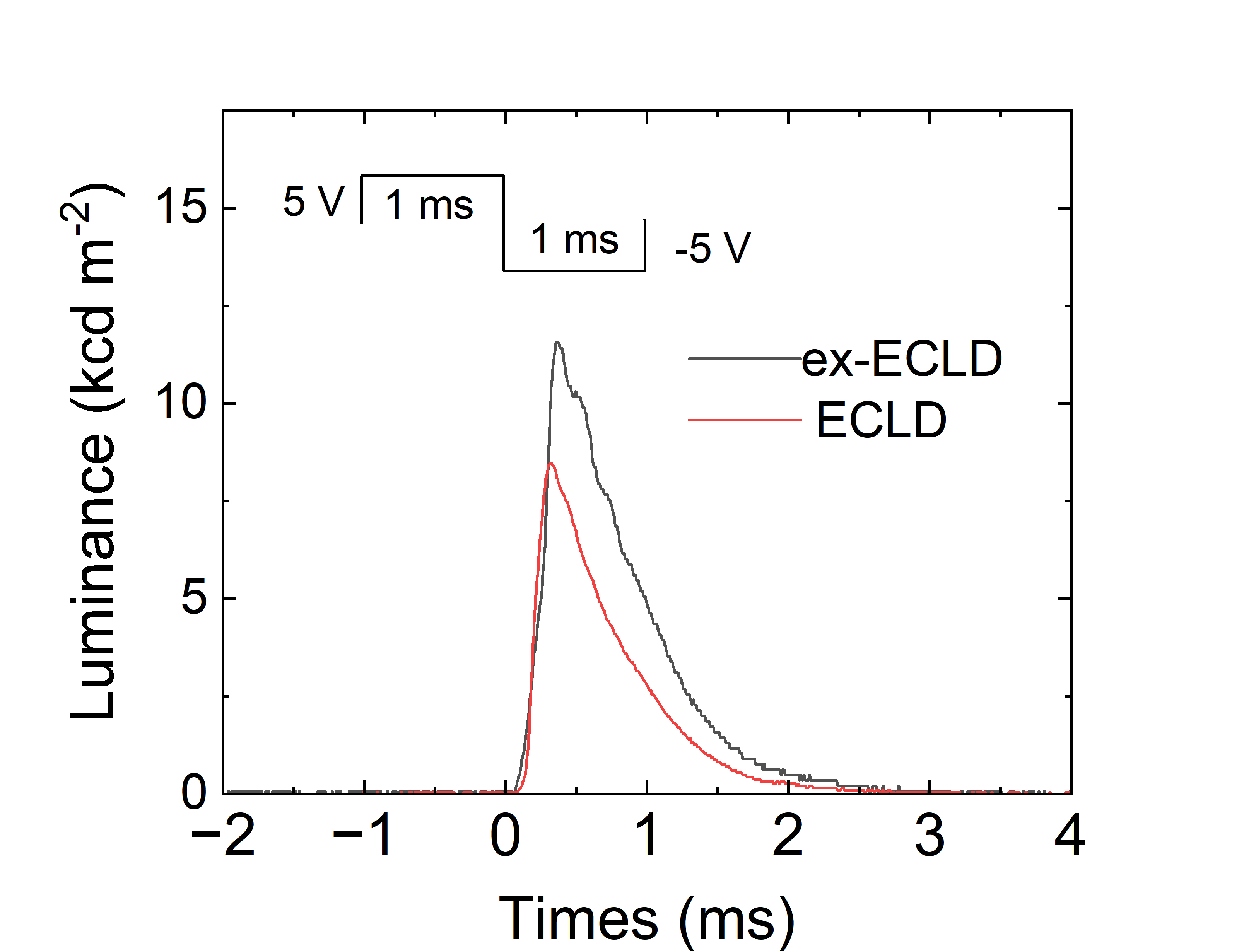


**Fig. S3** **Transient electrochemiluminescence (ECL) response of devices using exciplex materials in addition to emitter (black curve) and solely emitter (red curve).** The biphasic voltage sequence consists of +5 V for 1 ms followed by -5 V for another 1 ms. A decay with multiple small peaks over time is observed only from the device using exciplex materials, indicating that the multiple peaks result from long-range coupling of exciplexes.

**
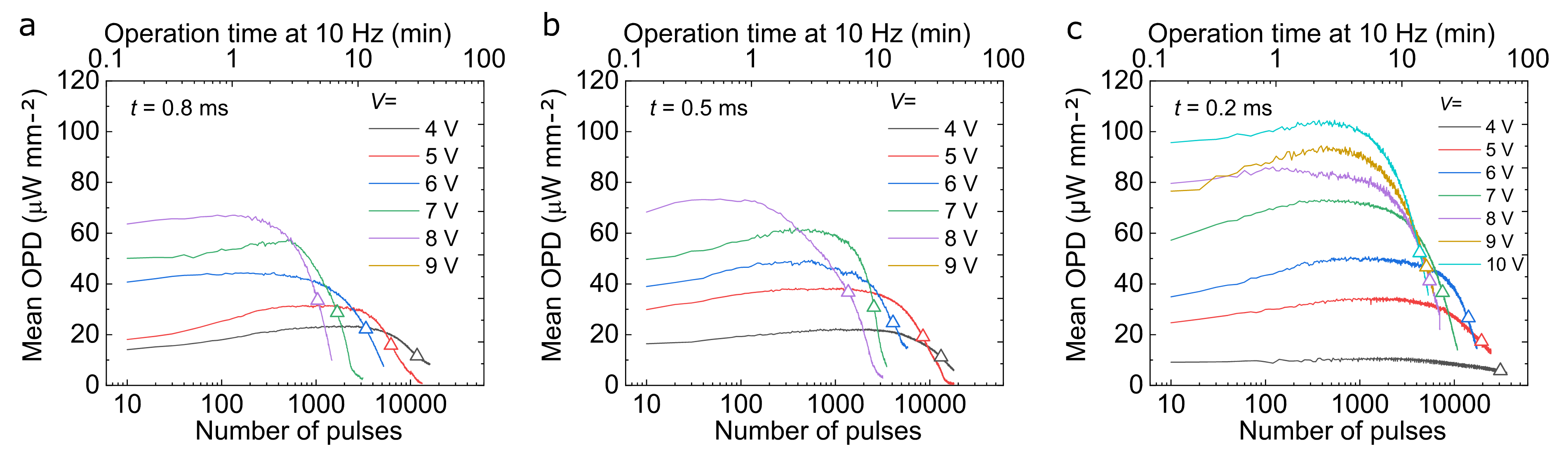
Fig. S4 Estimate of lifetime under pulsed operation.** **a-d** Mean optical power density (OPD) by ECL pulse versus number of given pulses for various voltages (*V*) ranging from 4 V to 10 V and various widths (*t*) ranging from 0.8 ms to 0.2 ms. All devices operated at a frequency of 10 Hz. Triangles represent the time points at which there is a 50% reduction to the maximum OPD during prolonged operation (LT_50_).


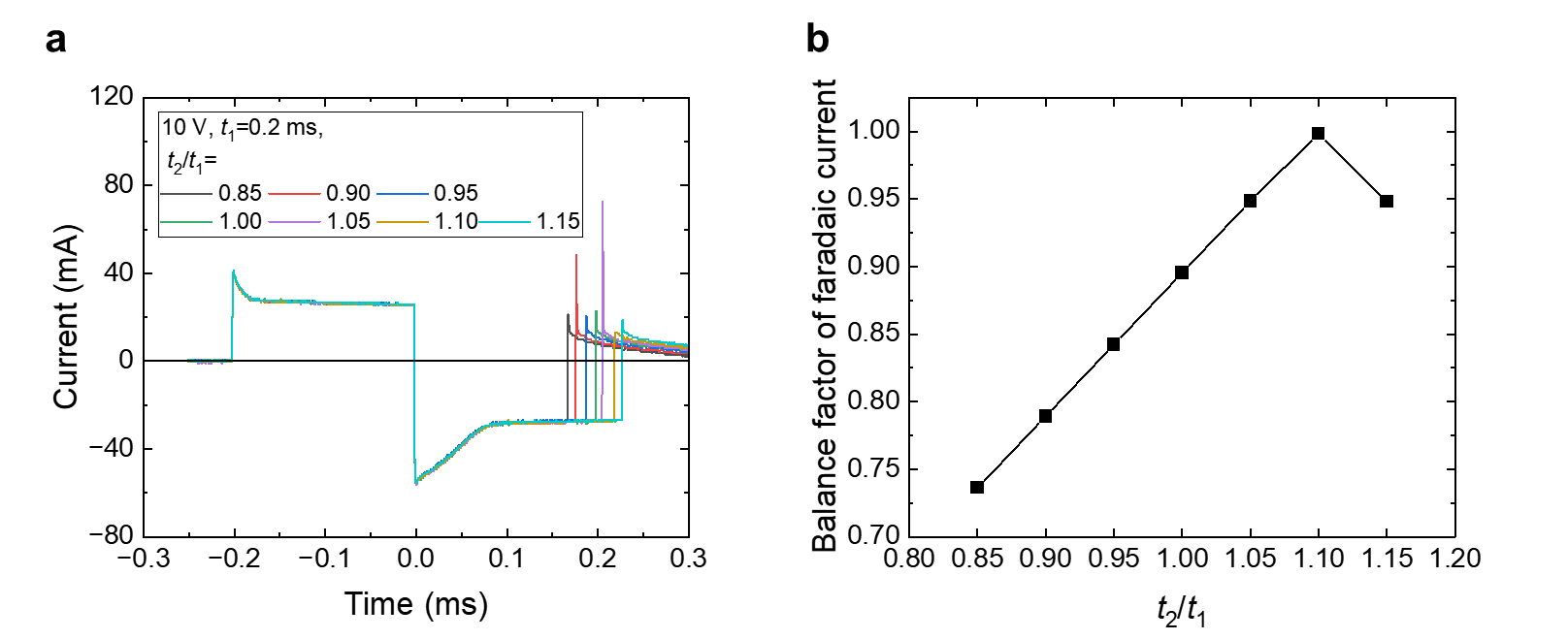


**Fig. S5 Balance factor estimation. a** Device current upon application of +10 V for 0.20 ms followed by -10 V for a time ranging from 0.17 ms to 0.23 ms. **b** Balance factor of faradaic current as a function of relative widths of the positive and negative voltage phases (*t*_2_/*t*_1_). A balanced injection of charges is measured at *t*_2_/*t*_1_=1.10.

**Fig. S6 Reduction in ECL peak intensity in 5th pulse depending on the rest period (*p*) between biphasic voltage sequences.** The voltage sequence consists of 10 V for 0.20 ms for the first phase and -10 V for 0.22 ms for the second phase.

**
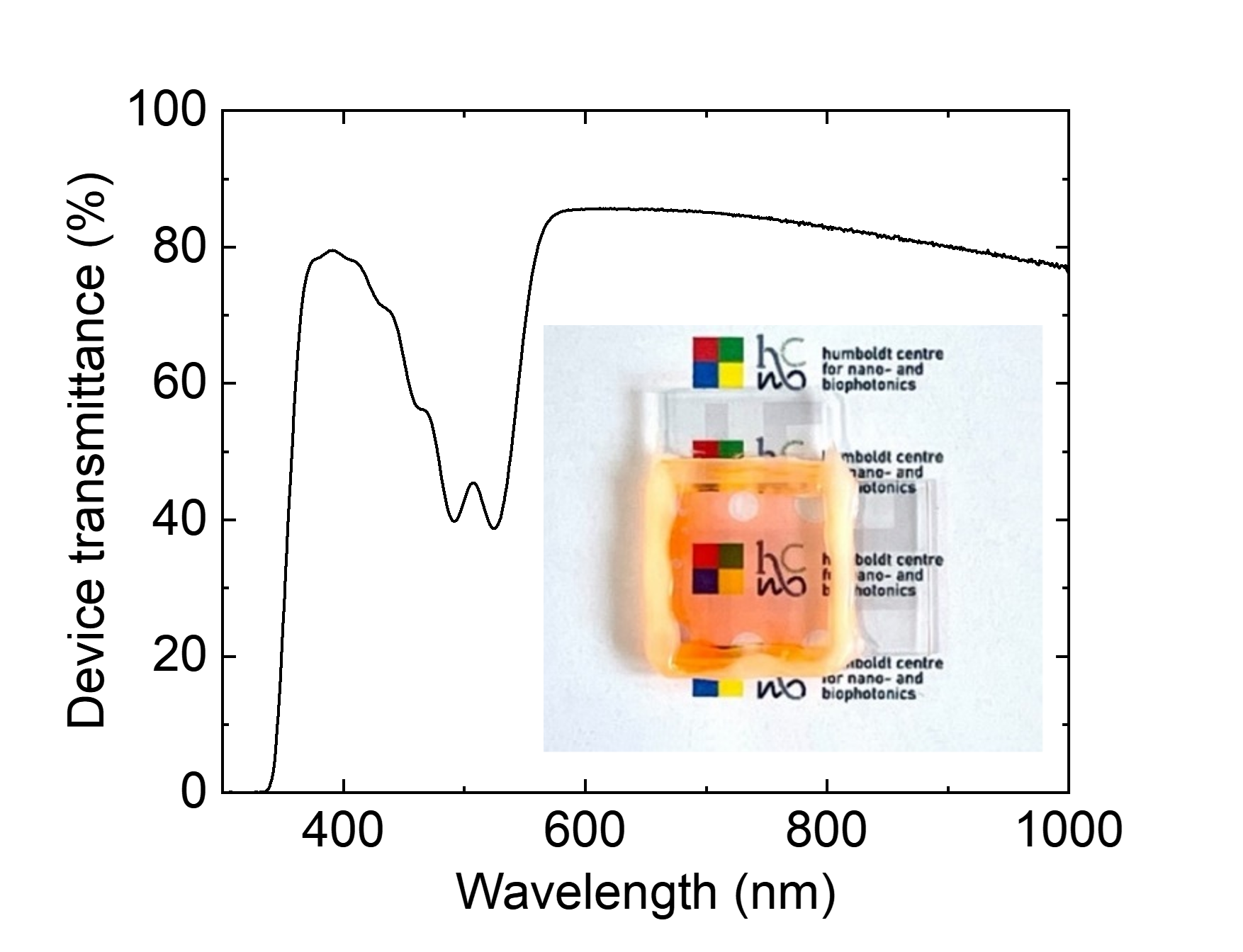
**

**Fig. S7 Transmittance of the ECLD measuring from 300 nm to 1000 nm.** Inset shows a photograph of the device on a white paper with printed logos of our research centre under illumination with fluorescent ceiling lights.


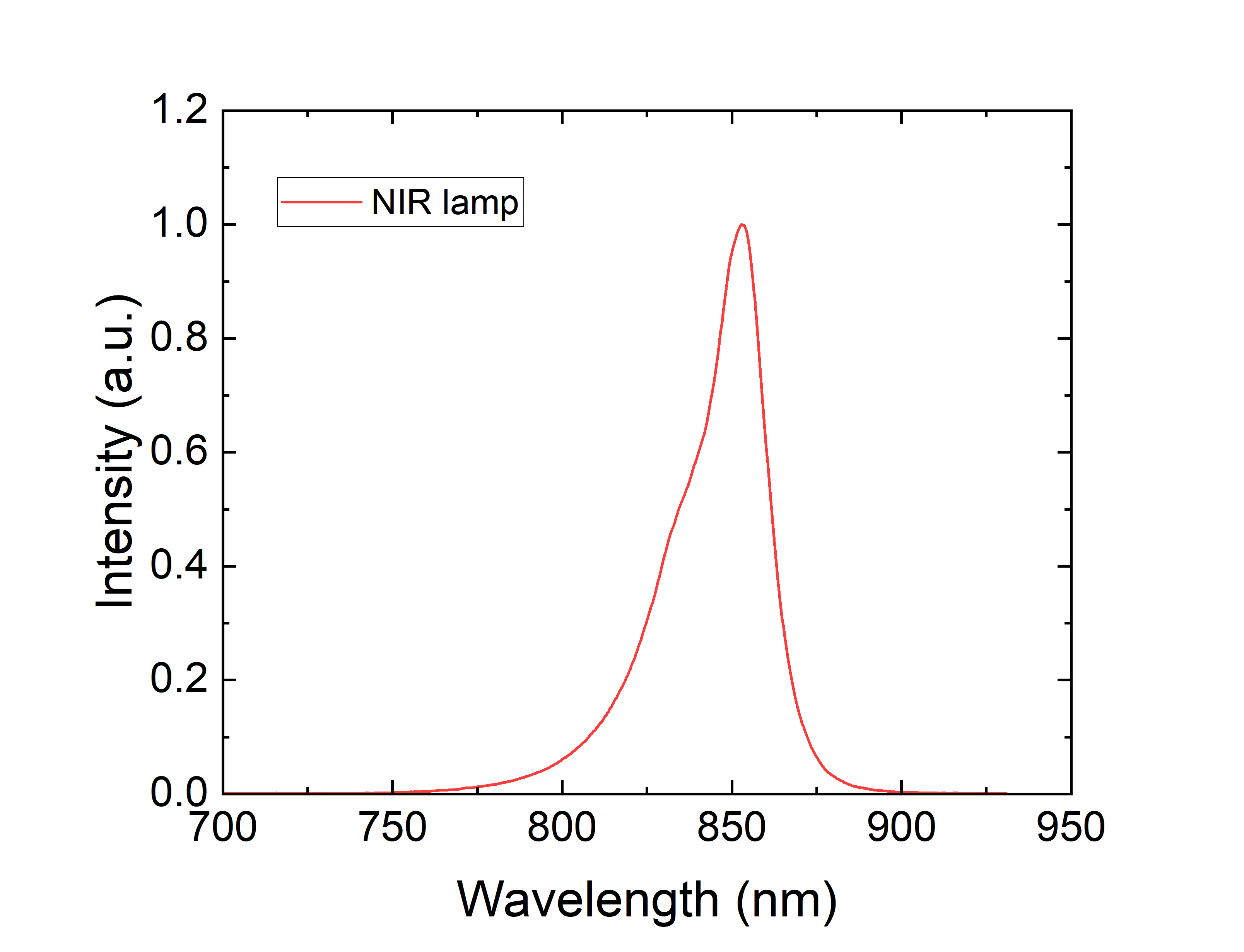


**Fig. S8 Infrared lamp spectrum used for the *Drosophila Larvae* experiments.**

**Fig. S9 Thermal imaging of ECLDs**. **a-b** Thermal images of ECLDs operated at *V*=10 V and 7 V, respectively, and with pulse trains consisting of *n*=1600 pulses at a 17% duty cycle. Device temperature was estimated by averaging over the 2×2 mm^2^ emissive area. **c-d** Evolution of absolute temperature and relative temperature change, respectively, during the active (on) and inactive (off) phases. The observed temperature rise of at most 0.2°C during ECLD operation is unlikely to impact the behavior of *Drosophila* larvae.**
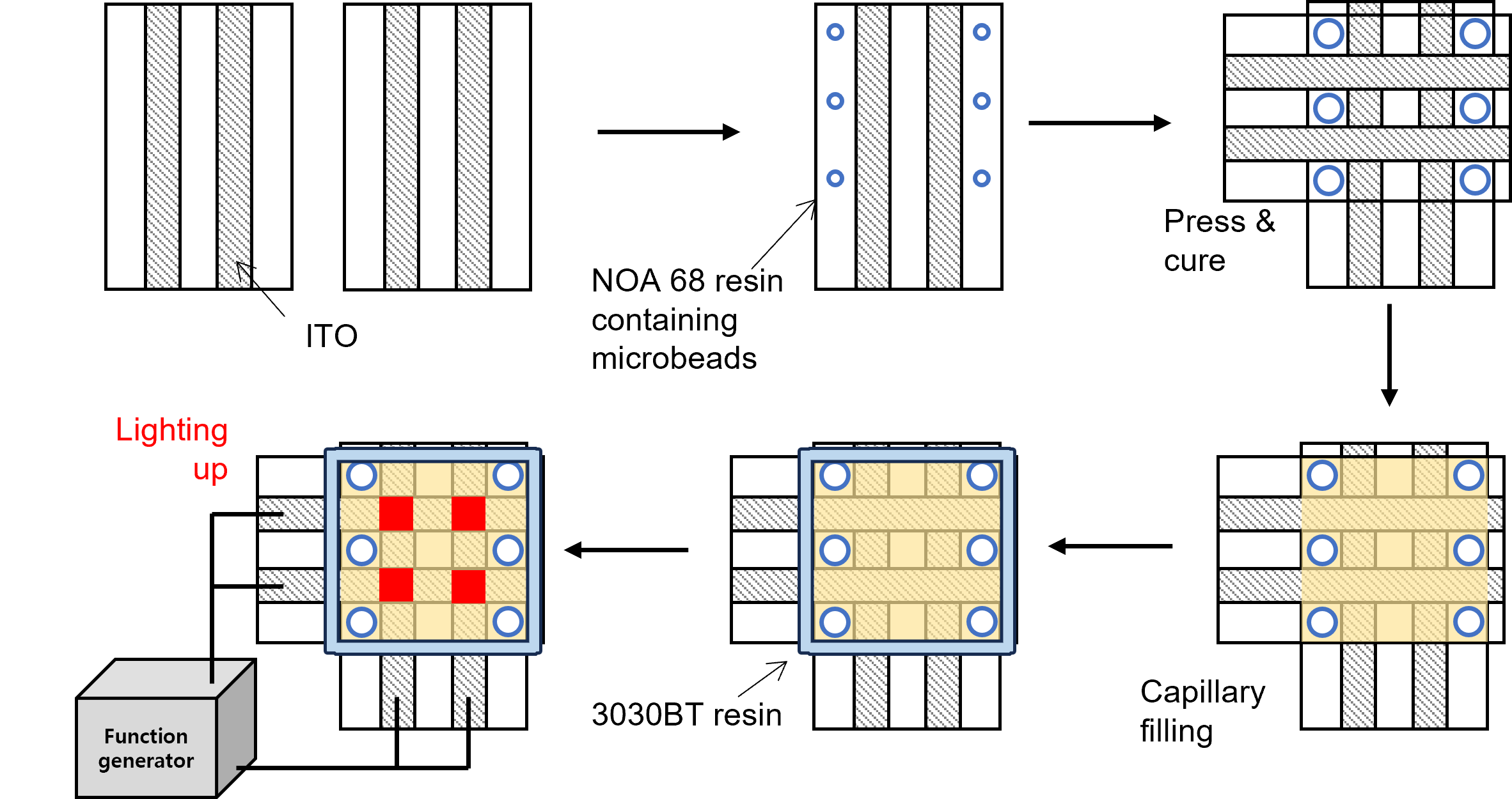
**

**Fig. S10 Fabrication procedure for an ECLD in a parallel electrode configuration.**
